# FOXM1 activates AGR2 and causes progression of lung adenomas into invasive mucinous adenocarcinomas

**DOI:** 10.1371/journal.pgen.1007097

**Published:** 2017-12-21

**Authors:** David Milewski, David Balli, Vladimir Ustiyan, Tien Le, Hendrik Dienemann, Arne Warth, Kai Breuhahn, Jeffrey A. Whitsett, Vladimir V. Kalinichenko, Tanya V. Kalin

**Affiliations:** 1 Division of Pulmonary Biology, the Perinatal Institute of Cincinnati Children’s Research Foundation, Cincinnati, Ohio, United States of America; 2 Abramson Family Cancer Research Institute, Perelman School of Medicine, University of Pennsylvania, Philadelphia, Pennsylvania, United States of America; 3 Thoracic Hospital at Heidelberg University, Heidelberg, Germany; 4 Institute of Pathology, University Hospital Heidelberg, Heidelberg, Nationales Centrum für Tumorerkrankungen (NCT) Heidelberg, Im Neuenheimer Feld, Heidelberg Germany; National Cancer Institute, UNITED STATES

## Abstract

Lung cancer remains one of the most prominent public health challenges, accounting for the highest incidence and mortality among all human cancers. While pulmonary invasive mucinous adenocarcinoma (PIMA) is one of the most aggressive types of non-small cell lung cancer, transcriptional drivers of PIMA remain poorly understood. In the present study, we found that Forkhead box M1 transcription factor (FOXM1) is highly expressed in human PIMAs and associated with increased extracellular mucin deposition and the loss of NKX2.1. To examine consequences of FOXM1 expression in tumor cells *in vivo*, we employed an inducible, transgenic mouse model to express an activated FOXM1 transcript in urethane-induced benign lung adenomas. FOXM1 accelerated tumor growth, induced progression from benign adenomas to invasive, metastatic adenocarcinomas, and induced SOX2, a marker of poorly differentiated tumor cells. Adenocarcinomas in FOXM1 transgenic mice expressed increased MUC5B and MUC5AC, and reduced NKX2.1, which are characteristics of mucinous adenocarcinomas. Expression of FOXM1 in Kras^G12D^ transgenic mice increased the mucinous phenotype in Kras^G12D^-driven lung tumors. Anterior Gradient 2 (AGR2), an oncogene critical for intracellular processing and packaging of mucins, was increased in mouse and human PIMAs and was associated with FOXM1. FOXM1 directly bound to and transcriptionally activated human *AGR2* gene promoter via the -257/-247 bp region. Finally, using orthotopic xenografts we demonstrated that inhibition of either FOXM1 or AGR2 in human PIMAs inhibited mucinous characteristics, and reduced tumor growth and invasion. Altogether, FOXM1 is necessary and sufficient to induce mucinous phenotypes in lung tumor cells *in vivo*.

## Author summary

Lung cancer is the leading cause of cancer-related deaths in men and women in the United States. It has a high rate of mortality due to late detection and aggressive metastasis. While multiple genes were implicated in the development of lung cancer, the molecular mechanisms that trigger the progression of benign lung adenomas to aggressive adenocarcinomas are still unclear. FOXM1 is a transcription factor, which is induced in lung cancers and is correlated with poor prognosis. In the present study, we used genetically-altered mice to express an activated form of FOXM1 protein in pre-existing benign lung adenomas. FOXM1 induced progression of lung adenomas into invasive, metastatic adenocarcinomas with a mucinous phenotype. In an orthotopic xenograft model of human mucinous lung adenocarcinoma, inhibition of FOXM1 suppressed mucinous characteristics and reduced the tumor invasion and metastasis. FOXM1 directly activated the *AGR2* gene, a key regulator of mucinous phenotype in lung cancer cells. We have demonstrated that FOXM1 is sufficient to drive progression of adenomas to adenocarcinomas and is nessesary to maintain a mucinous phenotype. Therapeutic targeting of FOXM1 can be beneficial for treatment of PIMA patients.

## Introduction

Lung cancers are classified into small cell lung cancer (SCLC) and non-small cell lung cancer (NSCLC). Adenocarcinoma, the most common subtype of NSCLC, is a complex disease harboring activating mutations in *KRAS* (30%), *EGFR* (15%), or *ALK* (5%) genes [1]. Recently, specific and effective receptor tyrosine kinase inhibitors have been generated to treat patients with *EGFR* and *ALK* mutations. Efforts to pharmacologically inhibit oncogenic KRAS, however, have been largely unsuccessful and developing targeted therapies for KRAS-driven lung cancer remains a significant challenge. While the majority of human NSCLCs have robust expression of NKX2.1, a homeobox transcription factor which is routinely used as a marker of NSCLC [2], a subset of the KRAS-driven tumors was identified with reduced expression of NKX2.1 [3, 4] associated with mucinous characteristics and poor prognosis in NSCLC patients [5]. NKX2.1 functions as a tumor suppressor in KRAS-driven mucinous adenocarcinomas, but has an oncogenic role in EGFR mutated lung tumors[3]. Haploinsufficiency of *Nkx2*.*1* in mice induced Kras^G12D^-mediated lung tumorigenesis and increased production of mucins in Kras^G12D^-driven lung tumors [3]. While NKX2.1 represses mucinous differentiation in NSCLCs, transcriptional activators of mucinous phenotype remain unknown.

Forkhead Box M1 (FOXM1) is a transcription factor activated by the RAS/ERK signaling pathway [6–8]. Activation of RAS-ERK drives cell cycle progression by regulating the temporal expression of cyclin regulatory subunits that bind to and activate their corresponding cyclin-dependent kinases (CDK). CDK2/Cyclin E and CDK1/Cyclin B complexes phosphorylate a variety of cell cycle regulatory proteins, including FOXM1, promoting G_1_/S and G_2_/M transitions [9, 10]. Activated ERK1/2, PLK1, CDK4 and CDK6 also phosphorylate FOXM1 and are required for full transcriptional competency of the FOXM1 protein [11, 12]. FOXM1 transcriptionally activates cell cycle regulatory genes critical for DNA replication and progression into mitosis, including *Cyclin B1*, *PLK1*, *JNK1*, *CDC25B*, *TOPO2* and *Aurora B* [13–16]. FOXM1 is required for KRAS/ERK signaling during lung morphogenesis, since deletion of *Foxm1* prevented defects in branching lung morphogenesis caused by *Kras*^*G12D*^ [6]. Deletion of *Foxm1* from respiratory epithelial cells blocked tumorigenesis by oncogenic *Kras*^*G12D*^ [8], and decreased the tumor initiation and growth of Kras-mutations-associated chemically-induced mouse lung tumors [17] [18]. In humans, increased FOXM1 was correlated with higher grades of lung cancers and poor patient survival [19]. While the role of FOXM1 in lung tumor initiation and growth is well established, it is unclear whether FOXM1 enhances progression from adenomas to adenocarcinomas and regulates metastasis of lung tumors *in vivo*.

In the present study, we used a doxycycline-inducible transgenic mouse model to express an activated FOXM1 transcript in pre-existing, benign lung adenomas. FOXM1 caused progression of lung adenomas into invasive, metastatic adenocarcinomas with a mucinous phenotype. Inhibition of FOXM1 in human mucinous adenocarcinoma cells inhibited mucinous characteristics and reduced tumor invasion in an orthotopic xenograft mouse model. We have demonstrated that FOXM1 is sufficient to drive progression of adenomas to adenocarcinomas and is required for maintainance of the mucinous phenotype.

## Results

### FOXM1 causes progression to invasive adenocarcinomas

**Fig 1:**
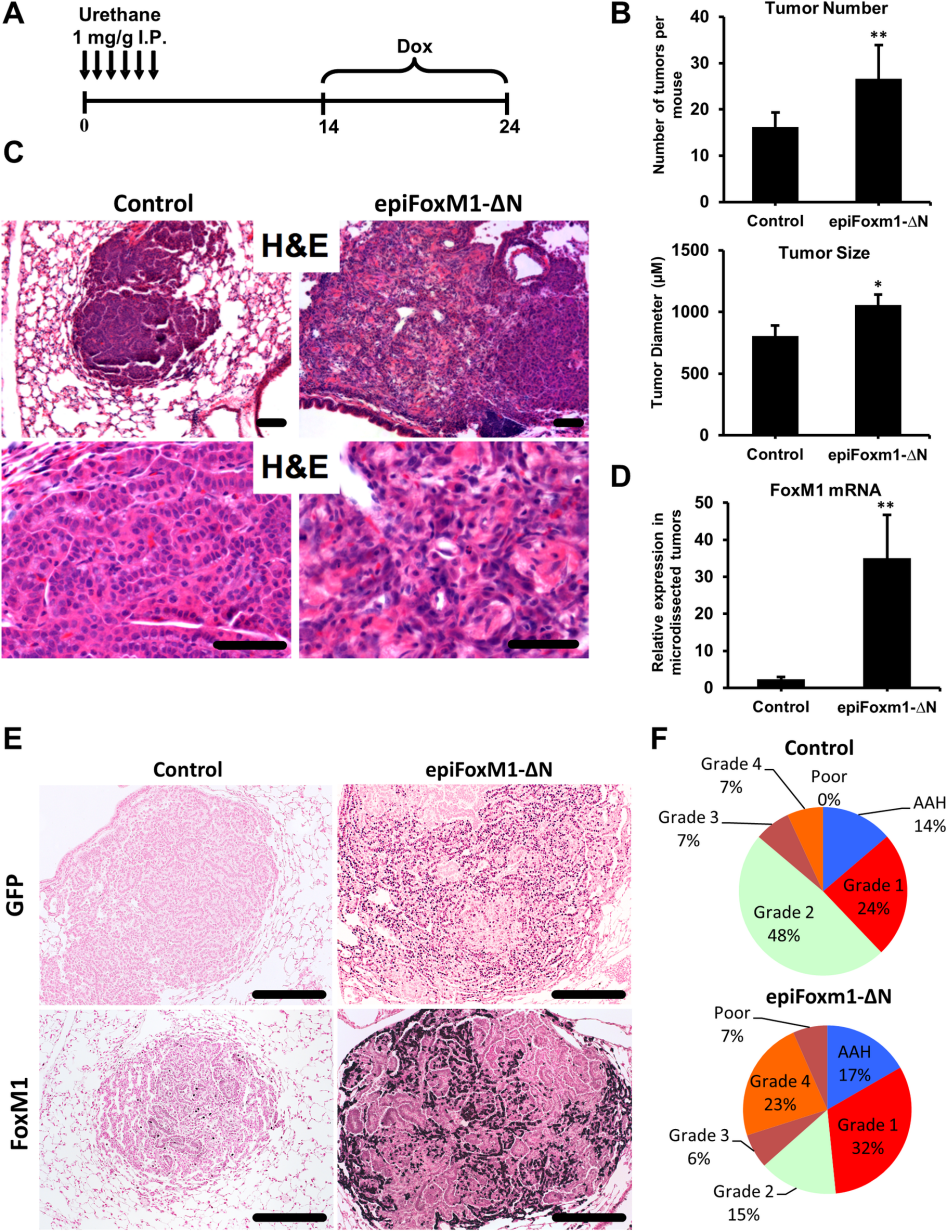
FOXM1 causes progression from benign lung adenomas to invasive adenocarcinomas. To induce lung adenomas, the *Spc-rtTA/tetO-GFP-FoxM1-ΔN* double transgenic (*epiFoxM1-ΔN*, n = 9) and control single transgenic (n = 6) mice were treated with six IP injections of urethane. At 14 weeks after the first urethane injection, when early low-grade lung adenomas were already present, *epiFoxM1-ΔN* mice were treated with Dox to induce *FOXM1-ΔN* transgene in SP-C-expressing tumor cells and epithelial type II cells. Mouse lungs were harvested at 24 weeks after the first urethane injection. **(A)**. Schematic of lung tumor induction and *FoxM1-ΔN* expression. **(B)** Average number (top) and size (bottom) of lung tumors following urethane treatment in control (n = 6 mice) and *epiFoxM1-∆N* (n = 9 mice) mice. **(C)** Representative H&E staining of lung tumors from control and *epiFoxM1-ΔN* mice demonstrates invasive and less differentiated phenotype of *epiFoxM1-ΔN* tumors compared to controls. **(D)** Efficient expression of *FOXM1-ΔN* mRNA in microdissected *epiFoxM1-ΔN* tumors (n = 5) compared to control tumors (n = 5) shown by qRT-PCR. mRNA levels were normalized to *β-actin* mRNA. **(E)** Efficient expression of transgenic FOXM1 protein in *epiFoxM1-ΔN* tumors shown by immunochistochemictry (IHC) using an anti-GFP antibody (top panels) and anti-FoxM1 antibody (bottom panels). **(F)** Schematic diagrams of the tumor grades distribution in control and *epiFoxM1-ΔN* mouse lungs. 25 tumors were analyzed from 9 *epiFoxM1-ΔN* mice, and 23 tumors from 6 control lungs. 10 images from each mouse lung were used for analysis. A p-value <0.05 is marked with a single asterik (*) and a p-value <0.01 is marked with a double asterik (**).

To examine the role of FOXM1 in pulmonary carcinogenesis, we used a urethane-induced lung cancer model. Mice containing *Spc-rtTA* and *tetO-GFP-FoxM1-ΔN* transgenes were generated. Treatment with doxycycline (Dox) expresses an activated form of FOXM1, the *FoxM1-ΔN*, tagged with GFP [20]. To induce lung adenomas, the *Spc-rtTA/tetO-GFP-FoxM1-ΔN* double transgenic mice (*epiFoxM1-ΔN*) were treated with urethane (Fig 1A). Low-grade lung adenomas were present 14 weeks after initiation of urethane [16, 21], at which time *epiFoxM1-ΔN* mice were treated with Dox to express the *FoxM1-ΔN* transgene in SP-C-expressing cells and lung epithelial cells. Lungs were harvested 10 weeks later for tumor assessment. Expression of activated FOXM1 increased the number and size of tumors (Fig 1B) and led to histologically less differentiated tumors shown with H&E (Fig 1C). qRT-PCR analysis of micro-dissected tumors demonstrated human (transgenic) *FOXM1* mRNA in *epiFoxM1-ΔN* lungs (Fig 1D). FoxM1-ΔN protein was detected in epithelial cells in the tumors by immunostaining (Fig 1E).

**Fig 2:**
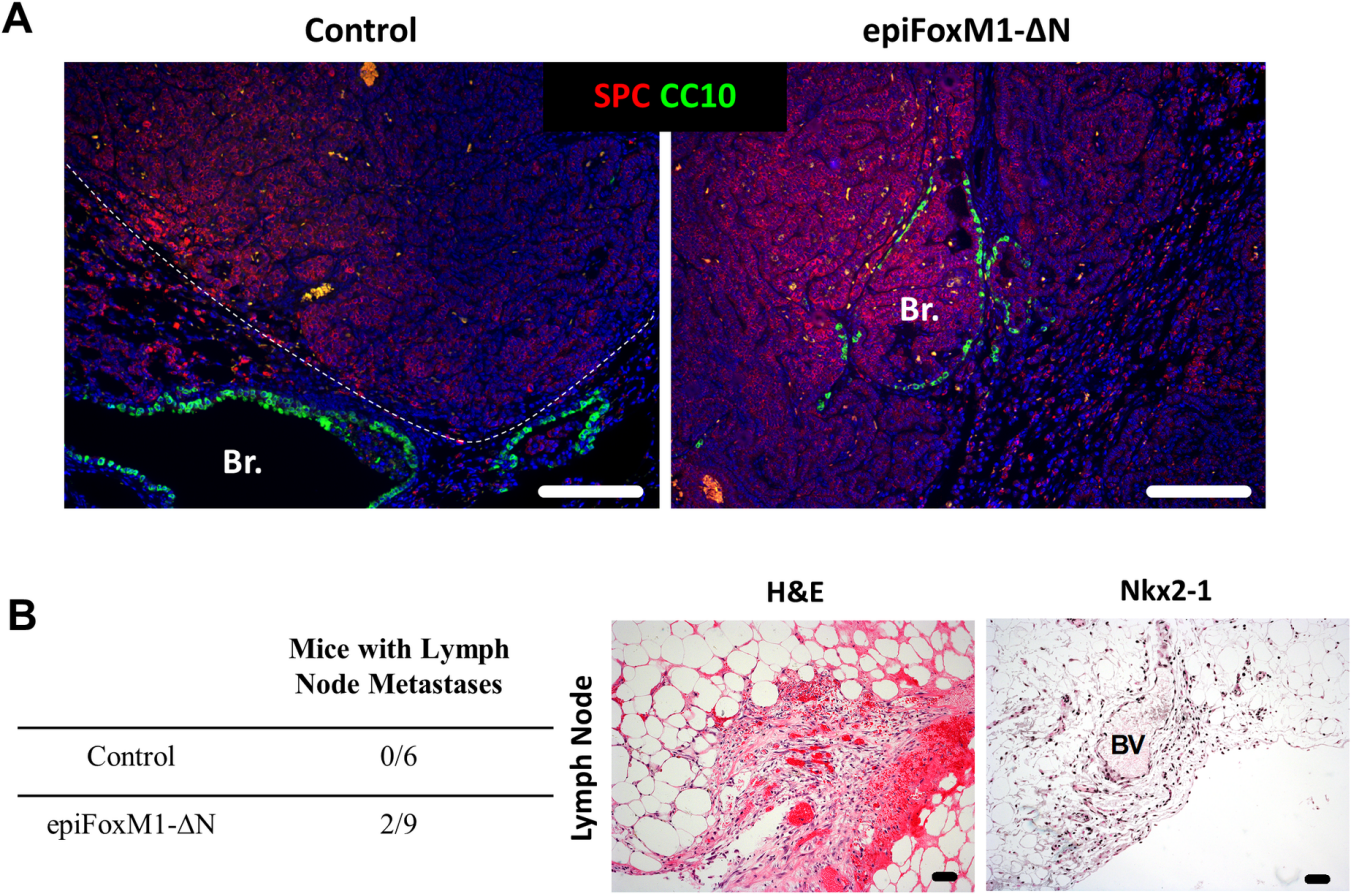
Expression of FOXM1 drives tumor invasion and metastasis. **(A)** Tumor invasion into the conducting airways was shown by immunofluorescence staining for pro-SPC (tumor cells, red) and CCSP (CC10, bronchiolar epithelium, green). **(B)** Frequency of peritoneal lymph node metastases (left panel). None of the control mice (n = 6) developed metastasis. Metastases were found in two *epiFoxM1-ΔN* mice (n = 9). H&E staining and NKX2.1 immunostaining of *epiFoxM1-ΔN* peritoneal metastasis are shown in right panels.

Consistent with published studies [21–23], the majority of tumors in control urethane-treated mice were classified as low grades 1 and 2 non-small lung tumors (72%). Only 14% of tumors were high grades 3 and 4 lung adenocarcinomas (Fig 1F and S1 Fig). Expression of *FoxM1-ΔN* in low-grade adenomas increased the frequency of highgrade 4 tumors (23% vs. 7%), and caused progression into poorly differentiated adenocarcinomas (7%) which were not detected in the controls (Fig 1F and S1 Fig). All tumors in control and *epiFoxM1-ΔN* mice were positive for proSP-C (Fig 2A), which is consistent with previous studies and confirms the type II alveolar epithelial origin of tumor cells [16, 24]. *EpiFoxM1-ΔN* tumors often invaded the airways as shown by proSP-C stained tumor cells in the bronchial lumen stained with CCSP (CC10) (Fig 2A). Peritoneal lymph node metastasis were found in *epiFoxM1-ΔN* mice which expressed lung-specific NKX2.1 (TTF-1) protein (Fig 2B, right panel), indicating that the metastases were derived from *FoxM1-ΔN*-positive tumors that expressed stabilized human *FOXM1* transgene [20]. Metastases were not present in control mice. Altogether, expression of activated *FoxM1-ΔN* in benign lung adenomas caused tumor progression into poorly differentiated, metastatic adenocarcinomas.

### FOXM1 increases cellular proliferation in *epiFoxM1-ΔN* tumors

**Fig 3:**
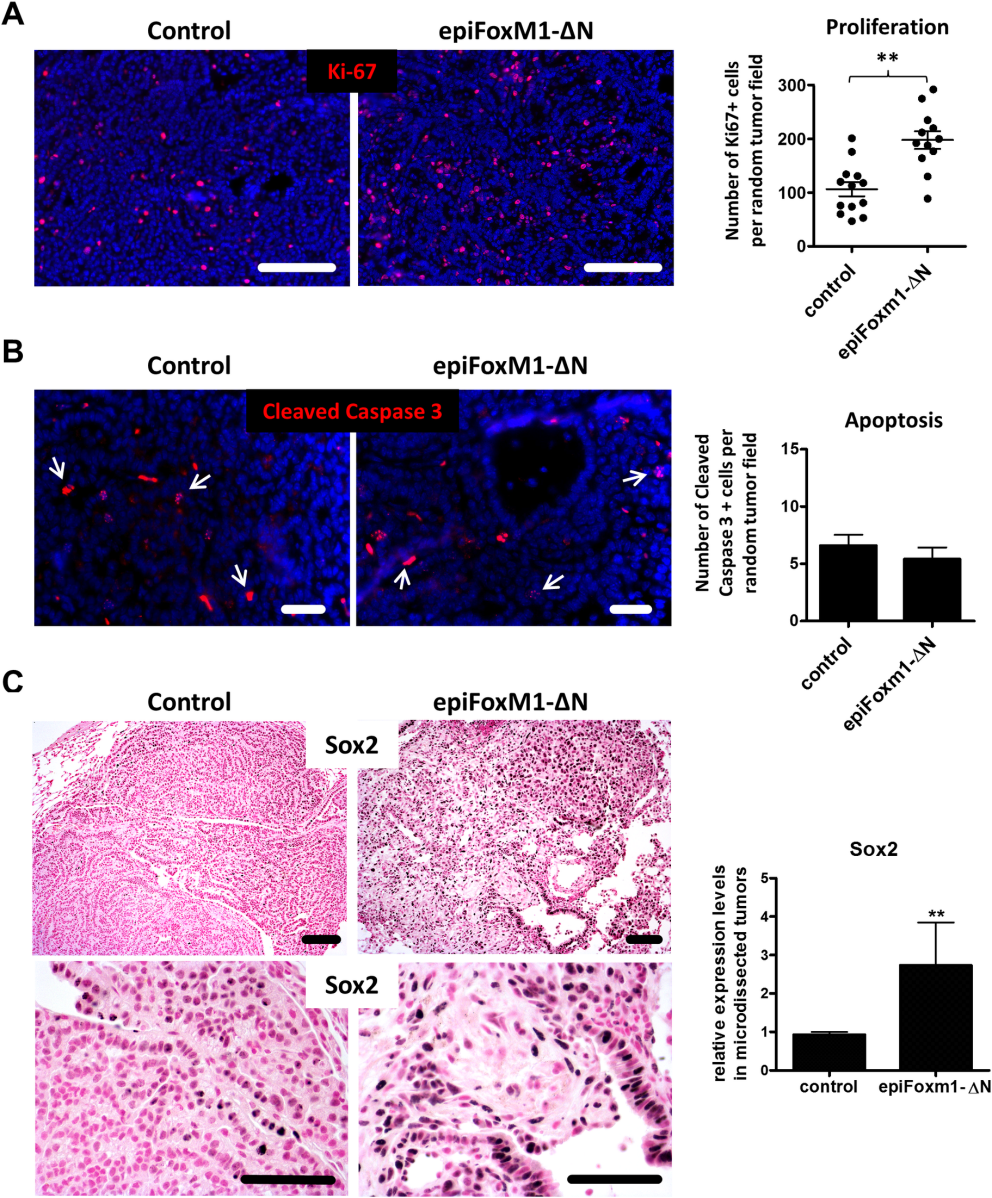
FOXM1 increases cellular proliferation in *epiFoxM1-ΔN* tumors. **(A)** Increased number of Ki67-positive cells in *epiFoxM1-ΔN* tumors is shown by immuno staining (left panels). Numbers of Ki67-positive cells were counted in ten random fields of control and *epiFoxM1-ΔN* tumors at 200x magnification (right graph). **(B)** No changes in apoptosis were found in *epiFoxM1-ΔN* tumors compared to controls. Tumors were stained with antibodies specific to cleaved caspase 3 (arrows, left panels) and the number of positive cells were counted (right graph). The number of cleaved caspase 3-positive cells was counted using ten random fields at 200x magnification. **(C)** Expression of FOXM1 in lung adenomas increased the number of SOX2-positive cells. The increased SOX2 protein is shown with immunohistochemistry using antibodies against SOX2 (left panels). Increased *Sox2* mRNA is demonstrated by qRT-PCR (right graph). *β-actin* mRNA was used for normalization. A p-value <0.01 is marked with a double asterik (**).

Since FOXM1 is often expressed in proliferating cells and activates cell cycle regulatory genes, we used immunostaining for Ki-67 to visualize proliferating cells. Tumors from *epiFoxM1-ΔN* mice had increased numbers of Ki-67-positive cells (Fig 3A). No differences were observed in the frequency of apoptosis, as assessed by cleaved-caspase 3 immunostaining (Fig 3B). SOX2, a marker of stem-like and less differentiated NSCLC tumor cells [25, 26] and a known transcriptional target of FOXM1 [27], was increased in the *epiFoxM1-ΔN* tumors (Fig 3C, left panels). *Sox2* mRNA was increased in micro-dissected lung tumors from *epiFoxM1-ΔN* mice (Fig 3C, right panel). Thus, expression of activated *FoxM1-ΔN* in lung adenomas increased tumor cell proliferation and induced SOX2.

### Mucinous characteristics of *epiFoxm1-∆N* tumors

**Fig 4:**
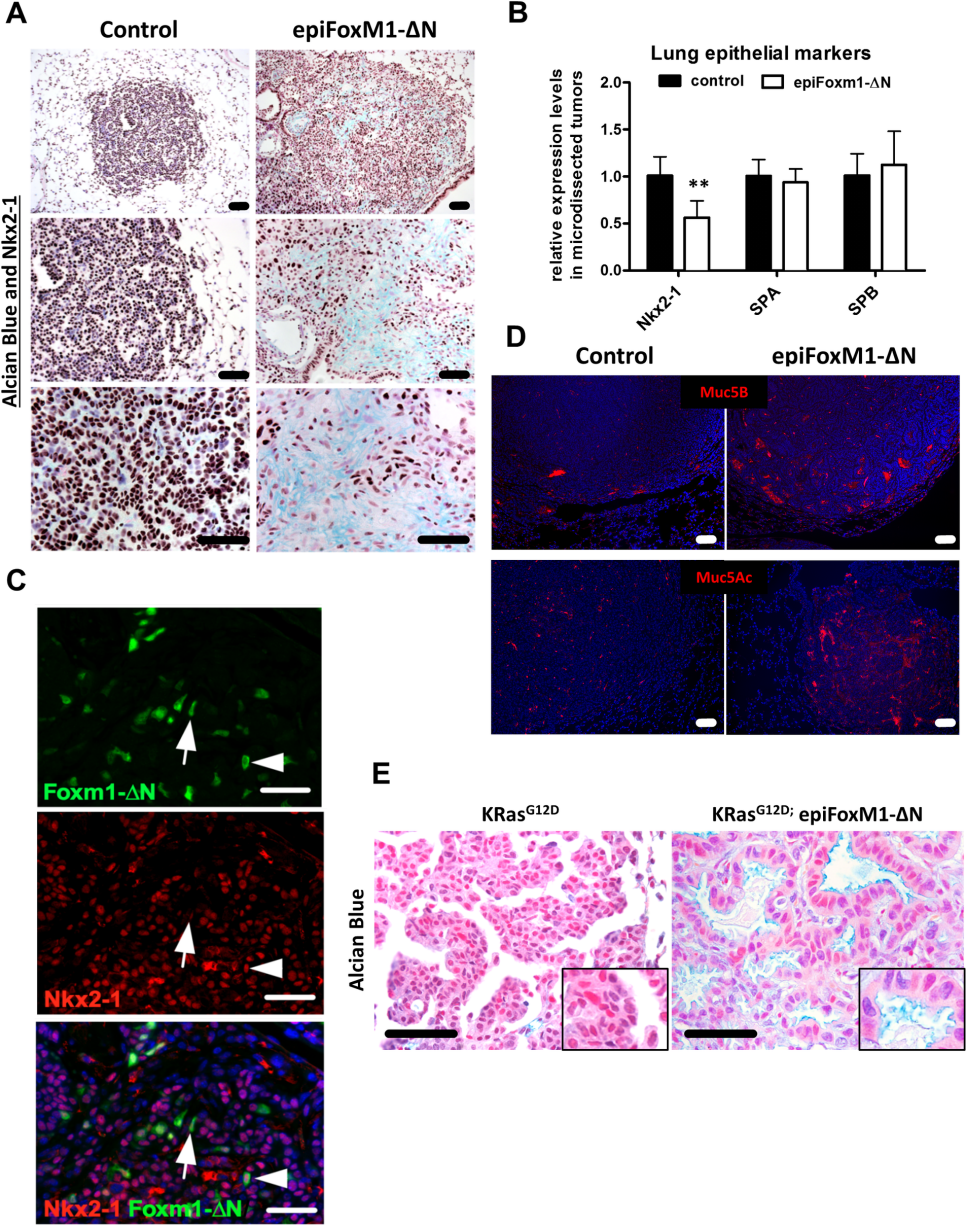
Expression of FOXM1 promotes mucinous characteristics in mouse lung tumors induced either by urethane or Kras^G12D^. **(A)** Lung sections form urethane-treated mice (control n = 6 and *epiFoxM1-ΔN* n = 9 mice) were stained with antibodies against Nkx2-1 and Alcian blue. Tumors in *epiFoxM1-ΔN* mice were highly positive for mucus (blue) and had low Nkx2-1 protein levels (brown). **(B)** *Nkx2-1* mRNA was decreased in microdissected *epiFoxM1-ΔN* tumors (n = 5) compared to control tumors (n = 5) as shown by qRT-PCR. *β-actin* mRNA was used for normalization. Data represent mean ± SD of three independent determinations using micro-dissected lung tumors from n = 6 control mice and n = 9 *epiFoxM1-ΔN* mice. **(C)** Co-localization studies demonstrated decreased Nkx2-1 in FoxM1-ΔN-positive tumor cells. **(D)** Tumors from epiFoxM1-ΔN mice stained positive for Muc5B and Muc5Ac as shown by immunofluorescence staining. **(E)** Parafin section from *Kras*^*G12D*^-induced lung tumors *(SPC-rtTA/TetO-Kras*^*G12D*^ mice, n = 3) and *Kras*^*G12D*^*; epiFoxM1-ΔN* mice (*SPC-rtTA/TetO-Kras*^*G12D*^*/TetO-FoxM1-ΔN* mice, n = 3) were stained with Alcian blue. FoxM1-ΔN caused mucus depositions in lung tumors induced with *Kras*^*G12D*^. A p-value <0.05 is marked with an asterik (*).

NKX2.1 is often used as a diagnostic marker of human NSCLCs [28], and its expression is decreased in mucinous tumor subtypes [3, 4]. While lung tumors from control mice maintained robust expression of NKX2.1, the NKX2.1 staining was decreased in *epiFoxM1-ΔN* tumors (Fig 4A, brown nuclei) and associated with decreased *Nkx2*.*1* mRNA (Fig 4B). NKX2.1 was absent in a majority of *FoxM1-ΔN*-expressing tumor cells (Fig 4C) and associated with increased extracellular mucus deposition as shown by Alcian Blue staining (Fig 4A). Expression of mucins MUC5B and MUC5AC were increased in *epiFoxM1-ΔN* tumors (Fig 4D). We also tested whether FOXM1 induced mucinous adenocarcinomas in a *Kras*^*G12D*^ model of lung cancer. Mice co-expressing both the *Kras*^*G12D*^ and the *FoxM1-ΔN* transgenes (*SPC-rtTA/ TetO- Kras*^*G12D*^*/ TetO-FoxM1-ΔN*, [20]) developed mucinous lung adenocarcinomas compared to mice expressing *Kras*^*G12D*^ alone (Fig 4E). Taken together, our data demonstrate that FOXM1 expression in lung tumors suppresses NKX2.1 and causes progression of lung adenomas into poorly differentiated, mucinous, metastatic lung adenocarcinomas.

### FOXM1 regulates AGR2 in mouse and human lung adenocarcinomas

**Fig 5:**
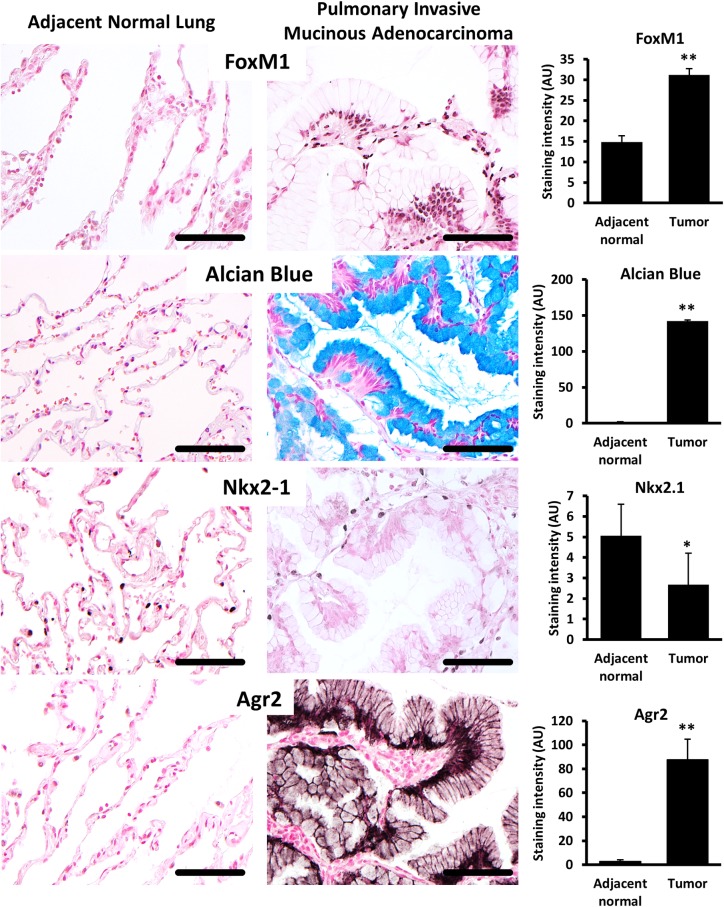
FOXM1 and AGR2 are highly expressed in human pulmonary invasive mucinous adenocarcinomas (PIMAs). Representative images of lung tissue sections from patients with PIMAs (n = 12 patients, S2 Table). Images include adjacent normal lung tissue (left panels) and tumor lesions (right panels) in matched patients. Tissue sections were stained with antibodies against FOXM1, AGR2, NKX2.1 or stained for mucus using Alcian blue (left panels). Image J software was used to quantify intensity of staining (right graphs). A minimum of 5 random 20x field images per patient were quantified. Increased FOXM1 staining in PIMAs was associated with abundant Alcian blue staining, loss of NKX2.1 and increased expression of AGR2 in tumor cells.

**Fig 6:**
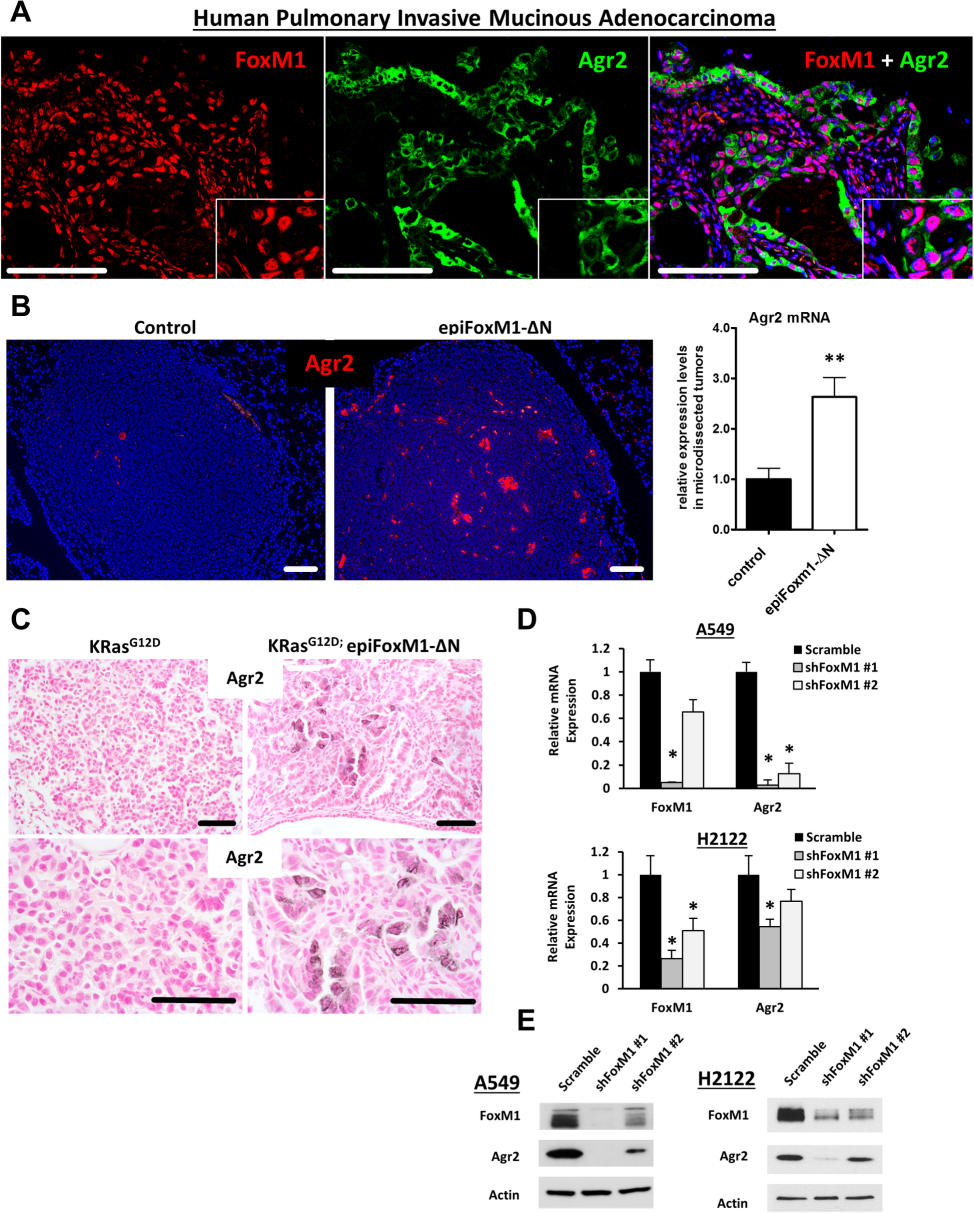
FOXM1 induces AGR2 in human and mouse lung adenocarcinomas. **(A)** In human PIMAs, immunofluorescence co-localization studies demonstrated that FOXM1-positive tumor cells were highly positive for AGR2. **(B**) In mouse urethane-induced lung cancer model, AGR2 was induced in *epiFoxM1-ΔN* tumors. Immunofluorescence staining for AGR2 in control and *epiFoxM1-ΔN* lung tumors (left panels, n = 6 control mice and n = 9 *epiFoxM1-∆N* mice, ten images per each mouse lung) and qRT-PCR of *Agr2* mRNA using RNA from microdissected control (n = 5) and *epiFoxM1-ΔN* (n = 5) lung tumors (right panel). *β-actin* mRNA was used for normalization. A p-value <0.01 is marked with a double asterik (**). **(C)** In mouse Kras^G12D^ –induced lung cancer models, AGR2 was induced in lung tumors of *Kras*^*G12D*^*/ epiFoxM1-ΔN* mice (n = 3) compared to control *Kras*^*G12D*^ mice (n = 3). **(D)** FoxM1 is required for AGR2 expression in PIMAs. Human PIMA cell lines A549 and H2122 were stable transduced with control (scramble) or shRNAs against human FoxM1 (shFoxM1 #1 or shFoxM1 #2). Efficient inhibition of *FoxM1* decreased *Agr2* expression in both A549 (top) and H2122 (bottom) PIMA cells, shown with qRT-PCR. *β-actin* mRNA was used for normalization. A p-value <0.01 is marked with a double asterik (**). **(E)** Western blot shows the correlation of the loss of FOXM1 and AGR2 in the A549 (top) and H2122 (bottom) cell lines.

FOXM1 staining was increased in tumor cells in human pulmonary invasive mucinous adenocarcinomas (PIMAs) compared to adjacent normal lung tissue, where FOXM1 was not detected (Fig 5A, upper panels). FOXM1 staining in human PIMAs was associated with abundant deposition of mucins (Fig 5A, middle panels), loss of NKX2.1 (Fig 5A middle panels) and increased expression of AGR2 (Fig 5A bottom panels), an ER chaperone critical for the posttranslational processing of mucins and associated with various oncogenic functions, [29, 30]. AGR2 was co-locolized with FOXM1 in human PIMA cells (Fig 6A). In mice, expression of *FoxM1-ΔN* in urethane-induced or Kras^G12D^-driven lung tumors led to increased AGR2 staining and *Agr2* mRNA expression (Fig 6B and 6C). Thus, FOXM1 induces AGR2 in mouse and human mucinous lung adenocarcinomas.

To determine whether FOXM1 regulates AGR2, we used shRNA lentiviral vectors to inhibit *FOXM1* expression in A549 cells, a human mucinous lung cancer cell line with a Kras^G12S^ mutation. Inhibition of FOXM1 reduced expression of AGR2 (Fig 6D and 6E) and was associated with decreased expression of proliferation-specific *Cyclins B1*, *E1*, *A1* and *D1* (S2A Fig). FOXM1 depletion reduced mRNAs of goblet cell associated genes *MUC5AC*, *MUC5B*, *MUC1* and *SPDEF* (S2B Fig), the latter is a critical transcriptional regulator of mucinous phenotype [31]. To demonstrate that the FOXM1 regulation of the mucinous phenotype is not limited to one cell line, we also inhibited FOXM1 in H2122 cells, another human mucinous lung adenocarcinoma with an activating mutation in Kras (Kras^G12C^). Similar to findings in A549 cells, inhibition of FOXM1 reduced *AGR2* mRNA and protein in H2122 cells (Fig 6D and 6E). Thus, FOXM1 is essential for AGR2 expression and the mucinous phenotype in human mucinous lung adenocarcinoma cell lines.

### FOXM1 induces transcription of *AGR2*

**Fig 7:**
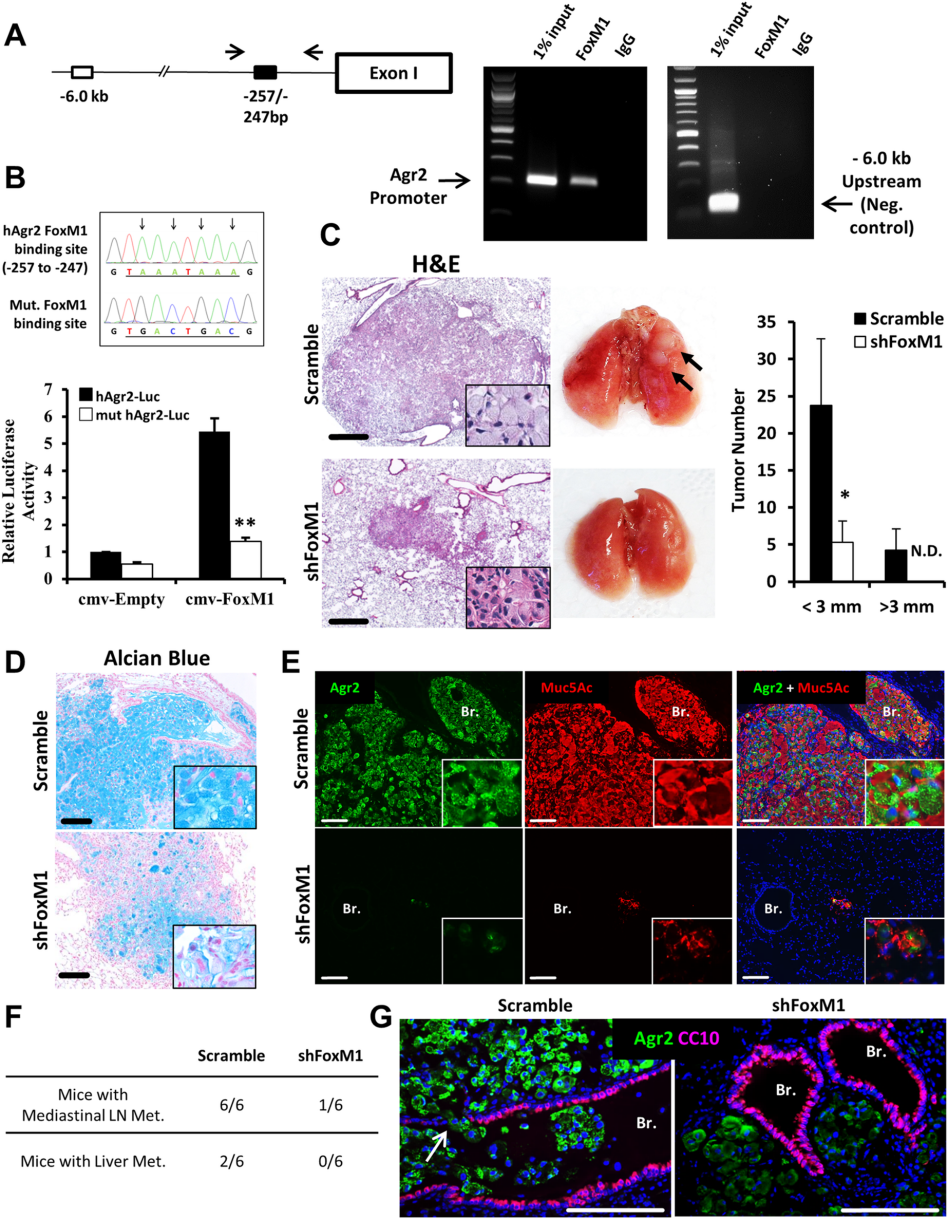
FOXM1 increases transcription of *AGR2* and is required to maintain mucinous phenotype in PIMAs. **(A)** Schematic of a potential FOXM1 binding site in the *AGR2* promoter (left panel). ChIP shows binding of FOXM1 to the *AGR2* promoter in A549 cells (right panel). A549 cells were fixed, lysed, sonicated, and used for immunoprecipitation with an antibody against FOXM1 or rabbit control IgG. PCR was performed encompassing a predicted FOXM1 binding site from -257bp to -247bp of the human *AGR2* promoter. DNA region located at –6.0 kb of *Agr2* promoter was used as a negative control. **(B)** FOXM1 transcriptionally activates *AGR2* promoter. Sequence denotes the predicted binding motif and the sequencing results following mutagenesis (top). Dual luciferase assay in Hek293T cells transfected with the WT (AGR2-Luc) or mutated -2.0kb *AGR2* (mut AGR2-Luc) promoter and an empty (CMV-Empty) or human FoxM1 expression (CMV-FoxM1) vectors is shown (bottom). A p-value <0.01 is marked with a double asterik (**). **(C)** Depletion of FOXM1 in human invasive mucinous A549 adenocarcinoma cells decreased lung tumor growth in the orthotopic xenograft model of lung cancer. A549 cells were inoculated into tracheas of immunocompromised mice. Representative images of H&E staining (5–8 images per mouse lung) and photographs of mouse lungs after eight weeks post tumor inoculation are shown (left panels). Tumor numbers and sizes in the two groups (n = 6 control (*Scramble*) and n = 6 *shFoxM1* mice) are shown (right panel). **(D-E)** FoxM1 is required to maintain mucinous phenotype in human PIMAs shown in orthotopic xenograft model of lung cancer (n = 6 control mice and n = 6 *shFoxM1* mice). **(D)** Inhibition of FOXM1 reduces mucus depositions shown with Alcian Blue, and **(E)** reduces AGR2 and MUC5AC expression in A549 orthotopic xenografts. AGR2 co-localizes with MUC5Ac in the orthotopic A549 xenografts. 5–8 images per mouse lung were used. **(F)** Frequency of observed mediastinal lymph node and liver metastases in control (*Scramble*) mice (n = 6) and *shFoxM1* mice (n = 6). **(G)** Control (*Scramble*) orthotopic A549 xenograft tumors (green) invade airways (purple). No invasion of tumor cells into airways was found in FOXM1-deficient tumors.

Since AGR2 was increased in *FoxM1-ΔN* expressing mouse lung tumors and human PIMAs (Fig 6), we tested whether FOXM1 directly activates the transcription of the *AGR2* gene. A potential FOXM1-binding site was found within the -257/-247 bp region of the human *AGR2* gene promoter (Fig 7A). Chromatin immunoprecipation (ChIP) in A549 cells demonstrated that FOXM1 directly bound to the -257/-247 bp region of the *AGR2* promoter (Fig 7A). Next we cloned a -2.0 kb fragment of the human *AGR2* promoter into luciferase (LUC)-expressing vector and co-transfected the -2.0 kb AGR2-LUC plasmid with a CMV-FOXM1 expression vector into A549 cells. FOXM1 increased the transcriptional activity of the *AGR2* promoter (Fig 7B). Site-directed mutagenesis of the FOXM1 binding site blocked the ability of CMV-FOXM1 to activate the *AGR2* promoter, indicating that the -257/-247 bp *AGR2* region was required for FOXM1-mediated activation of *AGR2* (Fig 7B). Thus, *AGR2* is a direct transcriptional target of FOXM1.

Interestingly, overexpression of AGR2 in FOXM1-defecient A459 cells rescued mRNA and protein levels of Cyclin D1, but was not able to rescue expression of other cyclins and mucinoius genes (Supplemental S3 Fig). Depletion of AGR2 alone was sufficient to decrease expression of proliferation-specific and mucin-specific genes (S3 Fig) and reduce the growth of A549 cell *in vitro* (S4A Fig). These data demonstrate that both FOXM1 and AGR2 are critical for cell growth and maintenance of the mucinous phenotype in PIMA cells *in vitro*.

### FOXM1 and AGR2 maintain mucinous characteristics of lung adenocarcinomas *in vivo*

We used lentiviral shRNA to stably inhibit FOXM1 in A549 cells in an orthotopic xenograft tumor model. Single cell suspensions of control and FOXM1-deficient A549 cells were delivered into the tracheas of immunocompromised mice. Eight weeks after inoculation, control A549 cells formed numerous tumors while FOXM1-deficient cells formed smaller and fewer tumors (Fig 7C). FOXM1-deficient xenografts were also less mucinous, with substantially reduced cytoplasmic swelling and reduced staining with Alcian blue (Fig 7C and 7D) and associated with decreased staining of AGR2 and MUC5AC (Fig 7E). Interestingly, parental A549 cells frequently metastasized into the mediastinal lymph nodes (6 out of n = 6 mice, 100% incidence of lymph node metastasis) and into the liver (2 out of n = 6 mice, 33% incidence of liver metastasis) (Fig 7F). In contrast, FOXM1-depleted tumors did not develop liver metastasis (0 out of n = 6 mice, 0%) and had decreased metastatic potential for mediastinal lymph nodes (1 out of n = 6 mice, 17%) (Fig 7F). In addition, control A549 tumors were locally invasive, infiltrating the pulmonary bronchioles and alveolar regions, whereas FOXM1-deficient A549 tumors were non-invasive (Fig 7G). Similar to intra-tracheal administration, a direct inoculation of FOXM1-deficient A549 cells into the left lung lobe inhibited the tumor growth and metastasis into the liver and mediastinal lymph nodes (S4B and S4C Fig). Similar effect was found after inhibition of AGR2 (S4B and S4C Fig). Thus, knockdown of FOXM1 or AGR2 reduced tumor invasion and inhibited mucinous phenotype of human lung A549 adenocarcinomas *in vivo*.

## Discussion

Activating KRAS mutations are associated with poor prognosis in patients with non-small cell lung cancers (NSCLCs), and KRAS mutant NSCLC tumors are often resistant to common anti-cancer drugs. Several KRAS-regulated kinases, including ERK1/2, PLK1, CDK1, CDK2, CDK4 and CDK6 phosphorylate FOXM1, contributing to its transcriptional activation. While FOXM1 alone was insufficient to induce lung tumors in transgenic mice [32], genetic deletion of *Foxm1* from mouse respiratory epithelium inhibited the initiation of lung tumorigenesis by Kras^G12D^ [8]. These results indicate that FOXM1 functions downstream of oncogenic KRAS to induce lung tumorgenesis. Deletion of *Foxm1* from fetal mouse lungs prevented the effects of activated Kras^G12D^ during lung development [6], demonstrating an important role for FOXM1 downstream of the KRAS/ ERK signaling cascade. In the present study, we found that FOXM1 is necessary and sufficient to induce mucinous characteristics in lung adenocarcinomas induced by either urethane or Kras^G12D^. The *FoxM1-ΔN*-expressing mouse tumors resembled human pulmonary invasive mucinous adenocarcinomas (PIMAs), an aggressive subtype of NSCLC associated with activating mutations in KRAS [1]. Consistent with these findings, we detected robust expression of FOXM1 in human PIMAs. Since FOXM1 is required for KRAS/ERK signaling in mouse tumor models, our studies provide a rationale for pharmacological targeting FOXM1 in PIMA tumors with activating KRAS mutations.

We found that transgenic expression of activated *FoxM1-ΔN* increased cell proliferation and tumor growth, findings consistent with the known role of FOXM1 in activation of cell cycle regulatory genes [13, 14]. Expression of FOXM1 in *Rosa26-FoxM1* transgenic mice accelerated proliferation of tumor cells and increased the number and size of lung adenomas after tumor initiation/ promotion with 3-methylcholanthrene (MCA)/butylated hydroxytoluene (BHT) [15, 32]. Likewise, genetic deletion of the *Foxm1* gene from adult mouse respiratory epithelium inhibited tumorigenesis caused by MCA/BHT or urethane [17]. In the present study, we observed abundant extracellular mucin and reduced NKX2.1 expression in *FoxM1-ΔN*-expressing tumors, features characteristic of human PIMAs [5]. This unexpected finding was further investigated using PIMA cell lines in which FOXM1 expression was required for expression of *MUC5AC*, *MUC5B*, *MUC1*, and the mucin-associated disulfide isomerase, *AGR2 in vitro* and in an orthotopic xenograft mouse model. Our data are consistent with published studies demonstrating that deletion of *Foxm1* from respiratory epithelial cells or pharmacological inhibition of FOXM1 by RCM-1 compound reduces MUC5AC and prevents differentiation of mucin-producing goblet cells in mouse asthma models [33, 34].

**Fig 8:**
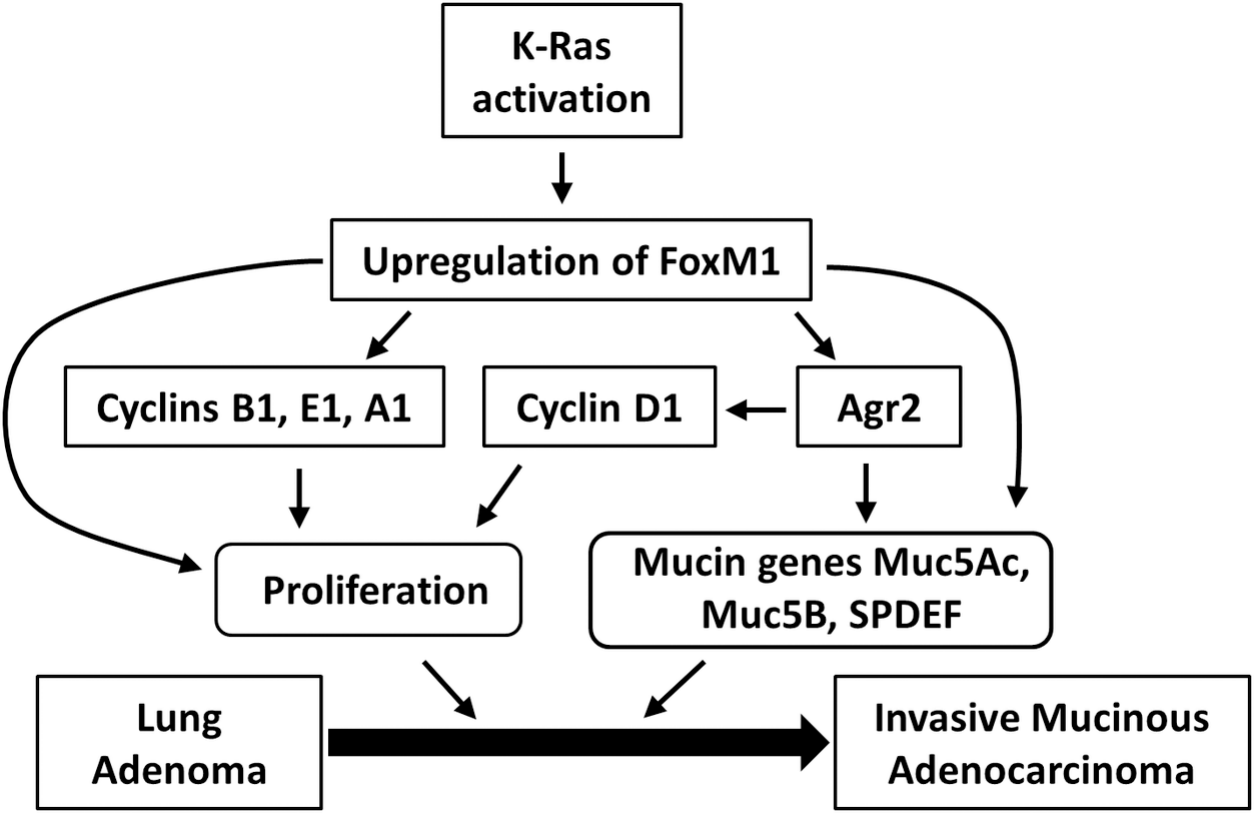
FOXM1 stimulates progression of lung adenomas into mucinous adenocarcinomas. Schematic drawing shows that FOXF1 induces expression of cell-cycle regulatory and mucinous genes, including *Agr2*, causing increased tumor cell proliferation and mucinous phenotype. FOXM1 directly activates transcription of *Agr2*. Both FOXM1 and AGR2 are critical for PIMA growth, invasion and progression of lung adenomas into aggressive mucinous adenocarcinomas.

AGR2 is an ER chaperone which functions as a proto-oncogene in breast, colon, and esophageal adenocarcinoma [30, 35–37]. Since AGR2 was highly expressed in the *Kras-LSL*^*G12D*^*; Nkx2*.*1-/+* mucinous lung carcinomas [3, 38], we investigated AGR2 expression in a carcinogen-induced *FoxM1-ΔN* transgenic mouse model and human PIMAs. We identified AGR2 was highly expressed in both PIMAs and *FoxM1-ΔN*-expressing mouse lung tumors, as a result of direct transcriptional activation by FOXM1. Although the oncogenic functions of AGR2 remain largely unknown, AGR2 expression was demonstrated to be prognostic in human lung cancer [39]. In normal goblet cells, AGR2 is required for folding and processing of secreted (MUC5AC) and membranous mucins (MUC1, MUC2, MUC5B;) which have also been implicated in various tumor-promoting processes [30, 40]. AGR2 was also shown to interact with reptin an inhibitor of p53, which may contribute to tumor cell proliferation and survival [41]. These molecular interactions may promote the mucinous and aggressive nature of PIMA tumors. Consistent with important role of FOXM1 and AGR2 in PIMAs, inactivation of either AGR2 or FOXM1 was sufficient to inhibit the tumor growth, invasion and metastasis in orthotopic mouse model. Altogether, our studies support a model in which FOXM1 functions downstream of KRAS and stimulates expression of AGR2 and other proliferation-specific and mucinous genes (Fig 8). Both FOXM1 and AGR2 induce tumor growth, progression, invasiveness and maintain mucinous characteristics in PIMAs. Since PIMA is a particularly aggressive cancer subtype without specific therapeutic options, our findings suggest that pharmacological inhibition of either AGR2 or its upstream regulator, FOXM1, may be beneficial for PIMA patients.

Modeling mucinous lung adenocarcinomas in mice requires both activation of oncogenic KRAS and downregulation of *Nkx2*.*1* [3]. Neither KRAS activation nor reduction of NKX2.1 alone is sufficient for carcinoma development or mucin production, indicating context specific crosstalk between these two genes. In the present study, expression of *FoxM1-ΔN* in urethane-induced lung adenomas decreased NKX2.1 and increased tumor progression and mucin production. Since urethane generates activating mutations in *Kras*, FOXM1 may regulate genetic interactions between *Kras* and *Nkx2*.*1* during development of mucinous lung adenocarcinomas. Interestingly, *FoxM1-ΔN*-expressing lung tumors contained many SOX2 expressing cells [27]. Since SOX2 is a marker of stem-like and dedifferentiated tumor cells [42], activation of SOX2 can contribute to increased invasiveness of lung tumors in *FoxM1-ΔN* transgenic mice.

In summary, activation of FOXM1 in pre-existed lung adenomas caused progression to invasive, metastatic adenocarcinomas. FOXM1 enhanced mucinous characteristics in lung tumors and transcriptionally activated *Agr2*, a mucin-associated oncogene. We have developed a mouse model of mucinous, metastatic lung adenocarcinomas and demonstrated that FOXM1 is necessary and sufficient to induce mucinous phenotype in lung tumor cells.

## Materials and methods

### Ethics statement

The cohort consisted of an independent lung adenocarcinoma patients who underwent lung cancer resection at the Department of Thoracic Surgery of the Thoraxklinik at the University Hospital Heidelberg. All samples were obtained with the informed consent of the patients and the approval of the local Ethics Committee of the University of Heidelberg (protocols 270/2001 and 206/2005). All animal studies were reviewed and approved by the Cincinnati Children's Hospital Medical Center Institutional Animal Care and Use Committee (protocol number IACUC2016-0070). We followed the guidelines outlined in CITI Training Program for Animal Care and Use (The Collaborative Institutional Training Initiative (CITI) and Association for Assessment and Accreditation of laboratory Animal Care (AAALAC).

### Animal models

*SPC-rtTA* mice harboring a 3.7kb fragment of the human SPC promoter was used to drive expression of an rtTA cassette as previously described [43]. *(TetO)*_*7*_*-CMV-GFP-FoxM1-ΔN* mice possessed a stabilized human *FOXM1* cDNA cassette fused to GFP as previously described [20]. *SPC-rtTA* mice were bred to *(TetO)*_*7*_*-CMV-GFP-FoxM1-ΔN* to generate *SPC-rtTA;TetO-FoxM1-ΔN* mice as previously described [20]. Mice with single or no transgenes were used as controls. *SPC-rtTA;TetO-FoxM1-ΔN* mice without doxycycline were also used as controls. 6–8 week old male mice received six intraperitoneal (I.P.) injections of urethane at a dose of 1 gram per kilogram of body weight. 14 weeks after the first injection, mice were maintained on chow containing doxycycline, except for *SPC-rtTA;TetO-FoxM1-ΔN* control mice. The *SPC-rtTA/ TetO- Kras*^*G12D*^*/ TetO-FoxM1-ΔN* mouse tumor model was used as previously described [20]. For A549 orthotopic xenograft experiments, 2.5x10^5^ A549 cells were inoculated into the tracheas or left lung lobes of 8 week old male NOD.*Cg-Prkdc*^*scid*^*;Il2rg*^*tm1Wjl*^/SzJ (NSG) immunocompromised mice.

### RNA isolation and real-time qRT-PCR

Total mRNA was isolated from microdissected lung tumors or cell lines using RNA Stat60 according to the manufacturer’s instructions. Isolated total mRNA was treated with DNASE I (Promega) according to the manufacturer’s instructions. DNASE treated RNA was reverse transcribed into cDNA using the High Capacity cDNA Reverse Transcription Kit (Invitrogen). Real time PCR was performed using the TaqMan Gene Expression Assays and a StepOnePlus Real-Time PCR System (Invitrogen) as described previously [44]. Inventoried TaqMan mouse gene assays are summarized in S1 Table. All reactions were performed in triplicate and normalized to *β-actin* mRNA.

### Lung histology, immunohistochemistry, and immunofluorescence

Mouse and human lung tissue paraffin sections (5μm) were stained with the following antibodies: anti-FOXM1 (Santa Cruz), anti-GFP (Abcam), anti-SPC (Seven Hills Bioreagents), anti-CC10 (Santa Cruz), anti-NKX2.1 (Seven Hills Bioreagents), anti-AGR2 (Cell Signaling), anti-MUC5Ac, anti-MUC5B (Abcam), anti-Ki67 (ThermoFisher Scientific), anti-Cleaved Caspase 3 (R&D Systems), anti-SOX2 (Santa Cruz). H&E Staining and Alcian Blue staining were performed according to the manufacturer’s protocol and as previously described [33, 45, 46]. Human lung tissue samples were provided by the tissue bank of the National Center of Tumor Diseases (NCT, Heidelberg, Germany) in accordance with the regulations of the tissue bank and the approval of the Ethics Committee of the Heidelberg University. Image J software was used for quantification of staining intensity in human lung tissue samples. Mean positive signal was quantified on 8-bit images with the following thresholds (low/high): FOXM1 (22/176), AGR2 (41/165), and NKX2.1 (58/160). For quantification of Alcian blue staining, the blue hue was quantified on color images using the following threshold settings: hue 136/143 pass, saturation 0/255 pass, and brightness 200/255 pass. A minimum of 5 random 20x field images per patient were quantified. Adjacent normal lung tissue was included only if clearly discernable in the patient sample.

### Generation of A549 and H2122 cell lines with stable knockdown of FOXM1

Plasmids expressing shRNA specific to human shFOXM1 #1 (5’-GCCAATCGTTCTCTGACAGAA-3’), human shFOXM1 #2 (5’-GCAAGAAGAAATCCTGGTTAA-3’), or shRNA control (5’-CAACAAGATGAAGAGCACCAA-3’) were used to generate lentiviruses. The stable knockdown of FOXM1 was achieved by transducing human lung adenocarcinomas A549 and H2122 with lentiviruses followed by puromycin selection as previously described [47].

### Western blot

Protein lysates were prepared from A549 and H2122 cell lines 48 hours after puromycin selection using RIPA buffer (Abcam) supplemented with 1mM PMSF (Sigma) and protease inhibitor cocktail (Roche). Western blot analysis was done as described previously [48, 49]. Primary antibodies were incubated overnight at 4°C in 1% nonfat, dry milk. β-actin was used as loading control. The following antibodies were used for Western blot: FOXM1 (Santa Cruz), AGR2 (Cell Signaling), or β-actin (Santa Cruz).

### Chromatin immunoprecipitation

A549 human lung adenocarcinoma cells were harvested, cross-linked using formaldehyde, sonicated to produce fragments approximately 500–1000 bp in size and immunoprecipitated using FOXM1 antibody or rabbit IgG antibody as previously described [50]. Revers cross-linked ChIP DNA samples were subjected to qPCR using oligonucleotides specific to promoter regions of human *AGR2*: -257/-247 (Fwd. 5’-TTGACAGGAGCAGGGAAGTATTGTAGA-3’; Rev.5’-CATTTGATTTGCCTGAAGGCTGATTTGT-3’). PCR products (expected size 211bp) were visualized by gel electrophoresis. A negative control region 6.0kb upstream of the *AGR2* transcriptional start site was selected for amplification (expected size 111bp) with the following primers: (Fwd. 5’-AGACCTCACCTTTTGTGTGC-3’; Rev. 5’-ACAAGCACAAGCCCATTCAC-3’).

### Cloning of the human *AGR2* promoter and dual luciferase assay

The human *AGR2* promoter region -1827bp to +197bp was amplified from A549 human genomic DNA using the following PCR primers: Forward-5’ CCTTGCCATCCGTCAGCCACTA 3’; Reverse- 5’TGCTGTCAGGAGCCTTACCTGG 3’. The PCR product was cloned into pCR2.1 TOPO and verified by DNA sequencing. The promoter fragment was subcloned into pGL2-Basic luciferase vector (LUC) by double digestion with Acc651 and XhoI restriction enzymes (New England Biolabs). For site-directed mutagenesis of the putative FOXM1 binding site, primers containing a 4 base pair change were used with the Site-Directed Mutagenesis kit (Invitrogen): Forward-5’ TGTGTGTCTTCAAGT***G***A***C***T***G***A***C***GGCAATCTGCCCACGGA 3’; Reverse-5’ TCCGTGGGCAGATTGCC***G***T***C***A***G***T***C***ACTTGAAGACACACA 3’. Hek293T cells were transfected with a CMV-Empty or CMV-human FOXM1b plasmids, as well as with LUC reporter driven by -2.0 kb *AGR2* promoter region (*AGR2*-LUC) WT or mutated AGR2-LUC promoter region. CMV-Renilla was used as an internal control to normalize transfection efficiency (relative luciferase activity). A dual luciferase assay (Promega) was performed 24 hours after transfection, as described previously [51, 52]. The standard error of the mean was calculated using the mean relative luciferase activity for each of the three wells in each group. All values were normalized to cells transfected with AGR2-LUC + CMV-Empty plasmids.

### Cloning of the human AGR2 cDNA

The human AGR2 cDNA was cloned from total RNA from A549 cells. RNA was isolated and reverse-transcribed into cDNA using random primers, as described above. For amplification of the AGR2 cDNA, we used Q5 High-Fidelity Polymerase (New England Biolabs) and the following primers: Forward-5’ ATGGAGAAAATTCCAGTGTCAG 3’; Reverse-5’ CTTTACAATTCAGTCTTCAGCAAC 3’. The 530bp band corresponding to the AGR2 cDNA was gel digested, cloned into PCR2.1 TOPO (Invitrogen) and sequence verified. The AGR2 cDNA was subcloned into BamHI and XhoI restriction sites in the retroviral plasmid MIEG3.

### Statistical analysis

Statistical analysis was performed using Prism software. ANOVA and Student’s T-test were used to determine statistical significance. Right skewed measurements were log-transformed to meet normality assumption prior to analysis. P values less than 0.05 were considered significant. Values for all measurements were expressed as the mean ± standard deviation (SD). Data were graphically displayed using GraphPad Prism v.5.0 for Windows (GraphPad Software, Inc., La Jolla, CA, USA).

## Supporting information

S1 Fig FOXM1 induces lung tumor progression. Representative H&E images depicting tumors of each grade observed in the lungs of control (n = 6) and *epiFoxM1-ΔN* (n = 9) mice. Scale bar = 500μM.
(TIF)

S2 Fig FOXM1 is essential for the expression of cell cycle regulatory genes and markers of mucinous tumors. mRNA isolated from control A549 (scramble) or shFoxM1 A549 stable cell lines were used for qRT-PCR. Knockdown of FOXM1 decreased mRNAs of cell cycle regulators (**A**) and mucinous characteristics (**B**) in A549 human pulmonary invasive mucinous adenocarcinoma. mRNA expression is normalized to β-actin mRNA. A p-value <0.05 is marked with a single asterik (*) and a p-value <0.01 is marked with a double asterik (**).
(TIF)

S3 Fig Expression AGR2 in FOXM1-deficient tumor cells does not restore the expression of cell cycle-regulatory genes and mucinous genes. (**A**) Western blot shows efficient overexpression of human Agr2 gene in Hek293T cells. (**B**) qRT-PCR analysis of proliferation-specific genes in stably transduced A549 cells. Only *Ccnd1* mRNA was restored to the control level after overexpression of AGR2. mRNA expression is normalized to β-actin mRNA. (**C**) Western blot shows the efficient knockdown of FOXM1 (shFoxM1) and overexpression of AGR2 in A549 cells. (**D**) qRT-PCR analysis of mucin markers. mRNA expression was determined by qRT-PCR and normalized to β-actin mRNA (n = 3 independent cell cultures). A p-value <0.05 is marked with a single asterik (*).
(TIF)

S4 Fig Deletion of FOXM1 or AGR2 decreased tumor cells growth in vitro and in orthotopic mouse model. (**A**) Growth of H2122 human mucinous lung adenocarcinoma cells was determined by MTT assay after knockdown of FOXM1 or AGR2. (**B**) Bioluminescent imaging of mice 35 days after orthotopic transplantation of A549 cells in the left lung lobe. Location of metastases are shown with arrows. (**C**) Tumor incidence and frequency of macroscopic metastases were determined 35 days after inoculation of A549 cells in the left lung lobe (n = 6 mice in each group).
(TIF)

S1 Table List of mouse and human Taqman probes used for experiments. (TIF)

S2 Table Clinical information of mucinous lung adenocarcinoma patient samples used in this study. (TIF)
