# Supplementary figures and images for: FOXM1 activates AGR2 and causes progression of lung adenomas into invasive mucinous adenocarcinomas
Source: PLoS Genet. 2017 Dec 21;13(12):e1007097. doi: 10.1371/journal.pgen.1007097 (PMC5755924; doi:10.1371/journal.pgen.1007097)

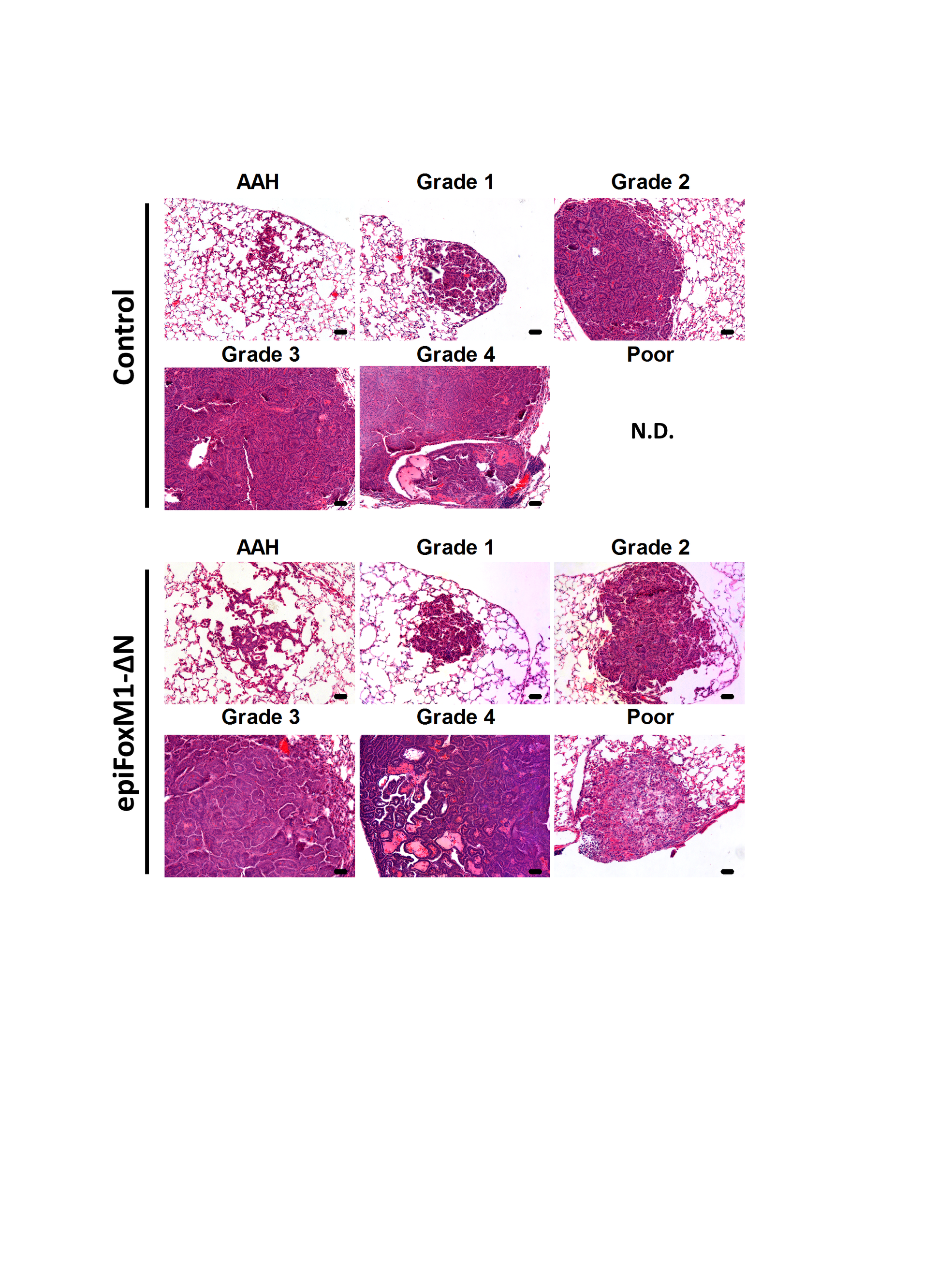

Supplement: S1 Fig — Representative H&E images depicting tumors of each grade observed in the lungs of control (n = 6) and epiFoxM1-ΔN (n = 9) mice. Scale bar = 500μM. (TIF) [file pgen.1007097.s001.tif]

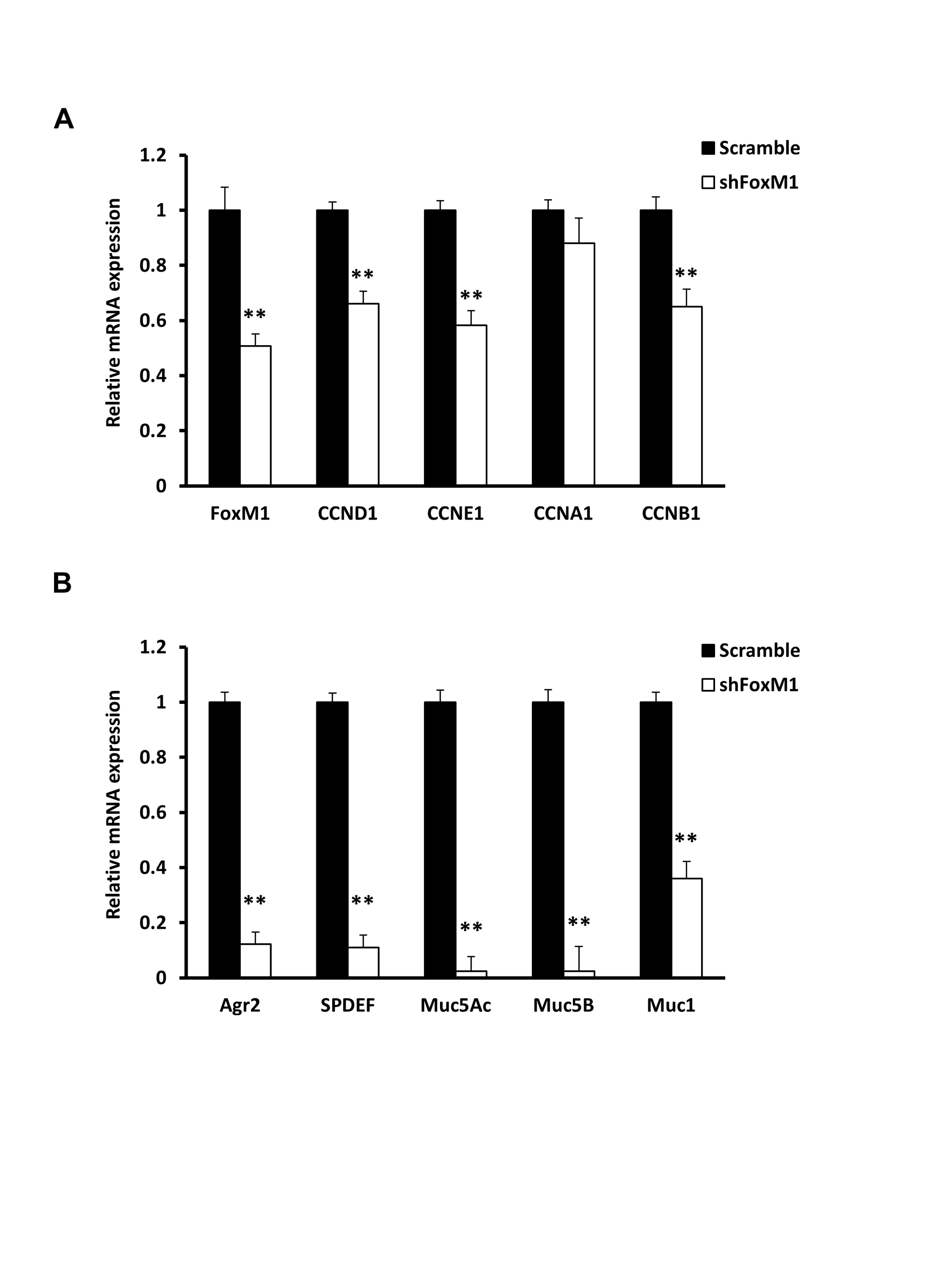

Supplement: S2 Fig — mRNA isolated from control A549 (scramble) or shFoxM1 A549 stable cell lines were used for qRT-PCR. Knockdown of FOXM1 decreased mRNAs of cell cycle regulators (A) and mucinous characteristics (B) in A549 human pulmonary invasive mucinous adenocarcinoma. mRNA expression is normalized to β-actin mRNA. A p-value <0.05 is marked with a single asterik (*) and a p-value <0.01 is marked with a double asterik (**). (TIF) [file pgen.1007097.s002.tif]

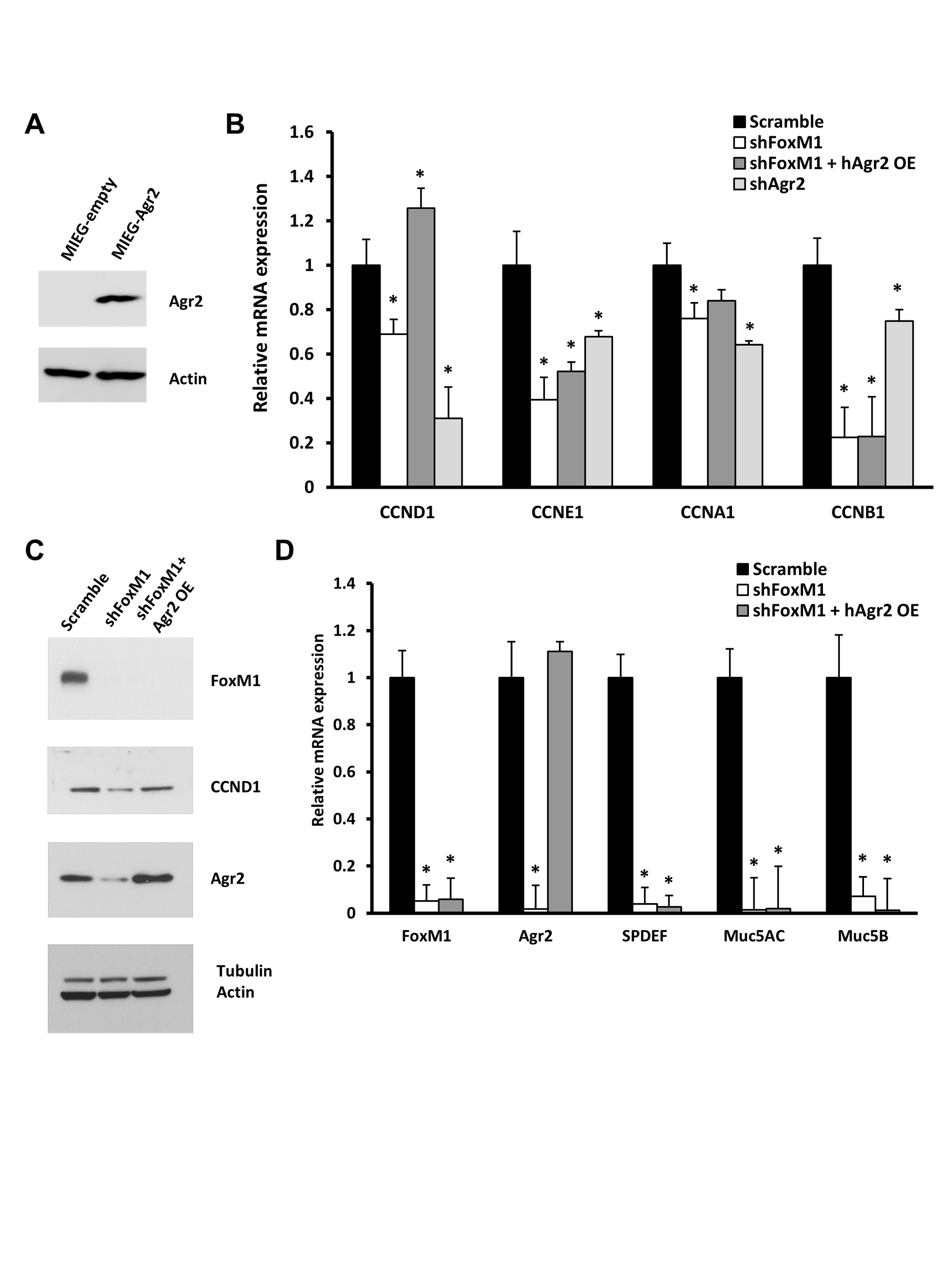

Supplement: S3 Fig — (A) Western blot shows efficient overexpression of human Agr2 gene in Hek293T cells. (B) qRT-PCR analysis of proliferation-specific genes in stably transduced A549 cells. Only Ccnd1 mRNA was restored to the control level after overexpression of AGR2. mRNA expression is normalized to β-actin mRNA. (C) Western blot shows the efficient knockdown of FOXM1 (shFoxM1) and overexpression of AGR2 in A549 cells. (D) qRT-PCR analysis of mucin markers. mRNA expression was determined by qRT-PCR and normalized to β-actin mRNA (n = 3 independent cell cultures). A p-value <0.05 is marked with a single asterik (*). (TIF) [file pgen.1007097.s003.tif]

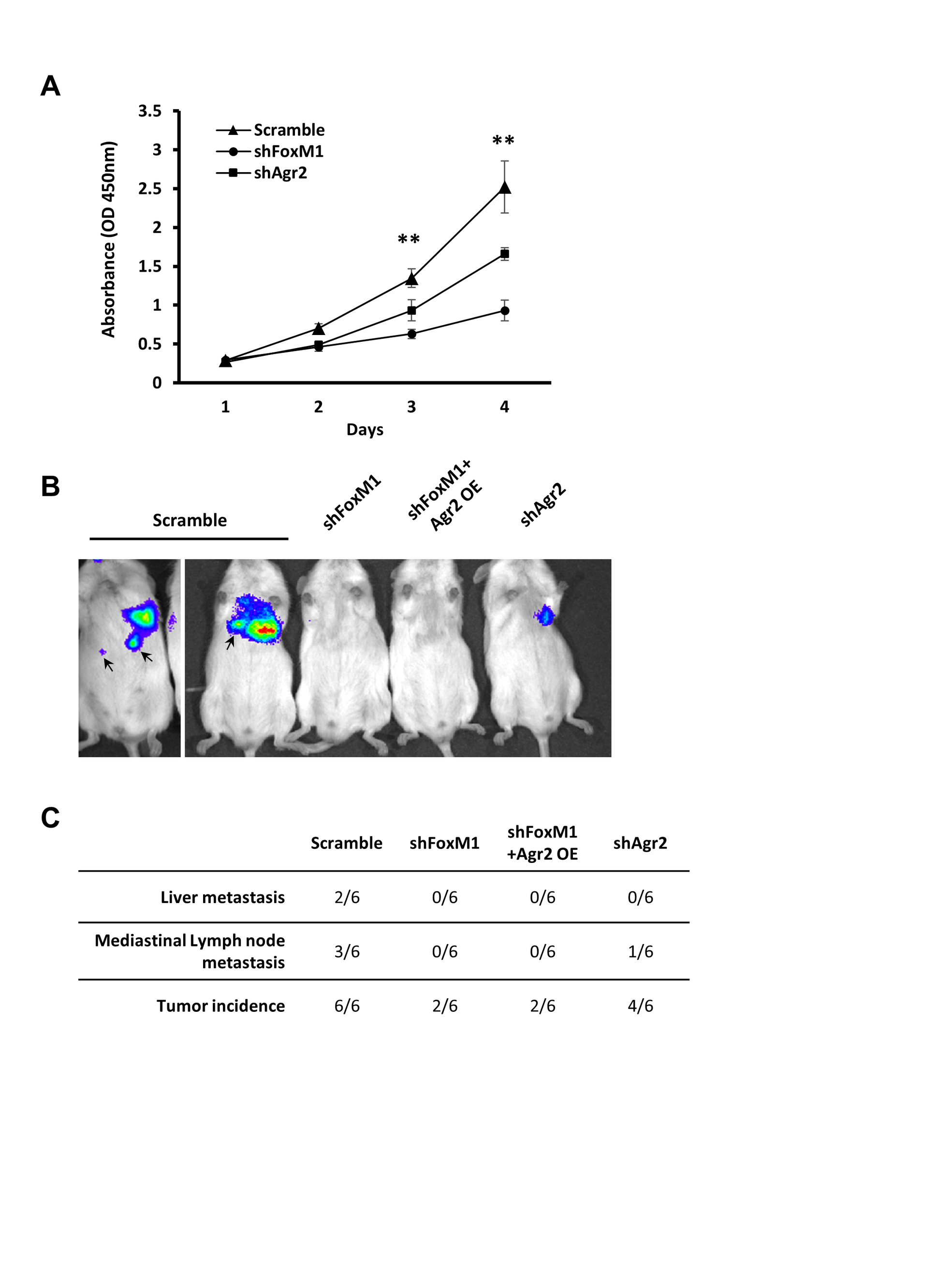

Supplement: S4 Fig — (A) Growth of H2122 human mucinous lung adenocarcinoma cells was determined by MTT assay after knockdown of FOXM1 or AGR2. (B) Bioluminescent imaging of mice 35 days after orthotopic transplantation of A549 cells in the left lung lobe. Location of metastases are shown with arrows. (C) Tumor incidence and frequency of macroscopic metastases were determined 35 days after inoculation of A549 cells in the left lung lobe (n = 6 mice in each group). (TIF) [file pgen.1007097.s004.tif]

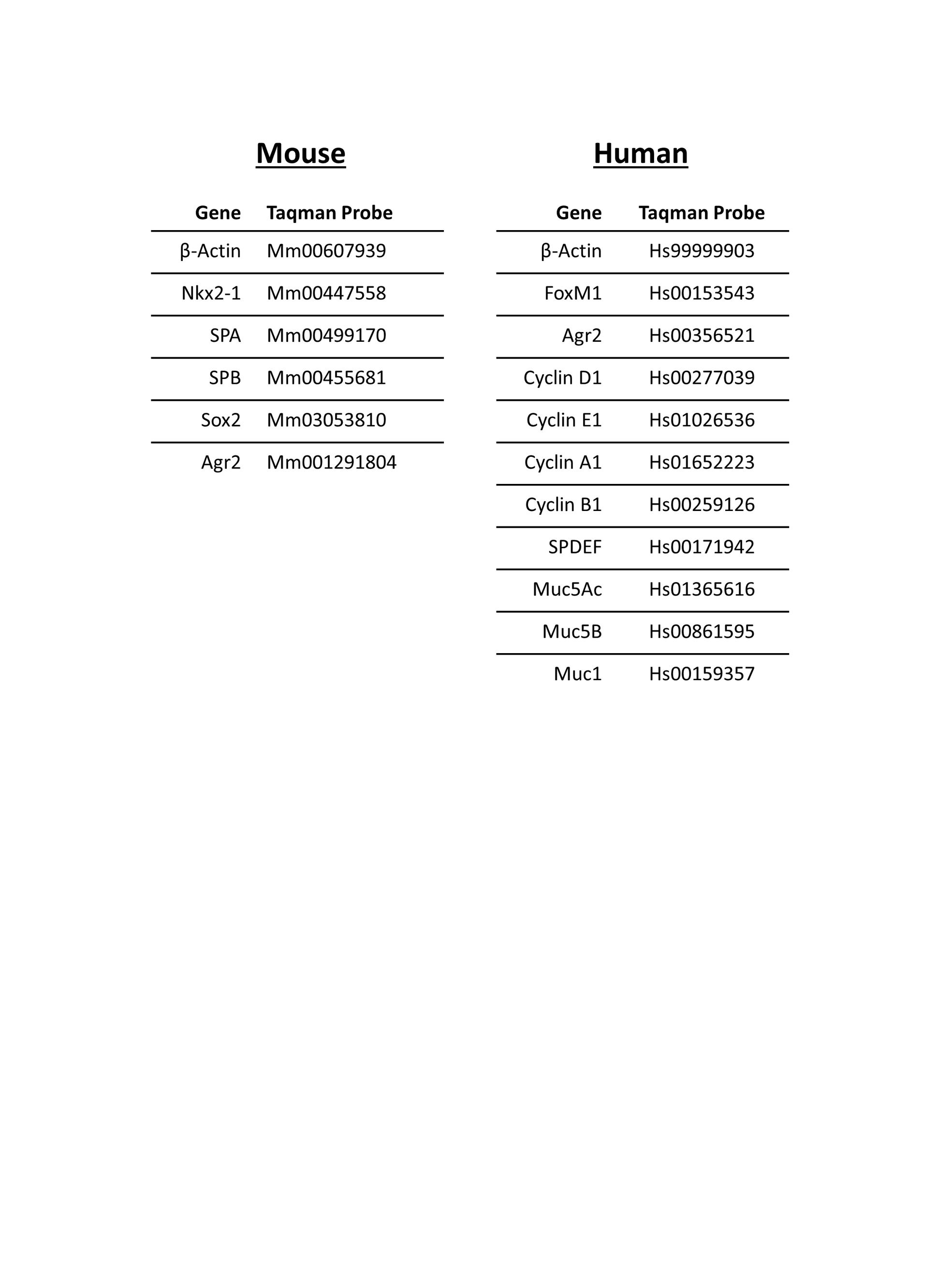

Supplement: S1 Table — (TIF) [file pgen.1007097.s005.tif]

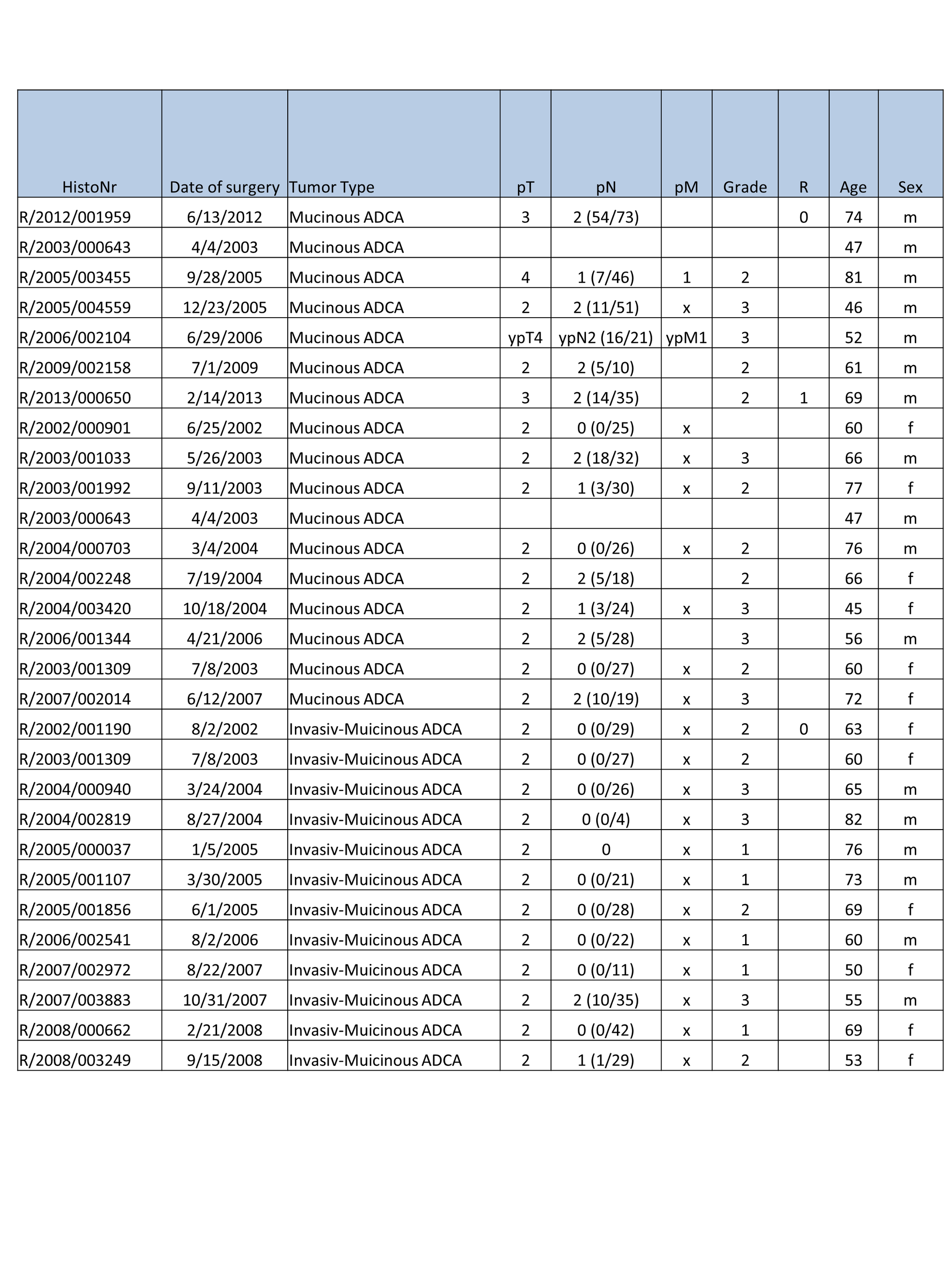

Supplement: S2 Table — (TIF) [file pgen.1007097.s006.tif]
